# Assessment of Youth-Friendly Service Quality and Associated Factors at Public Health Facilities in Southern Ethiopia: A Facility-Based Cross-Sectional Study

**DOI:** 10.1155/2019/9696278

**Published:** 2019-05-23

**Authors:** Betebebu Mulugeta, Meseret Girma, Gemechu Kejela, Feleke Gebre Meskel, Eshetu Andarge, Eshetu Zerihun

**Affiliations:** ^1^Amref Health Africa, Arba Minch, Ethiopia; ^2^Department of Public Health, College of Medicine and Health Science, Arba Minch University, P.O. Box 21, Arba Minch, Ethiopia

## Abstract

**Background:**

Evidence shows that services for youths are poorly coordinated and uneven in quality. There is a lack of evidence which informs the level of youth-friendly service quality in the study area. So, this study fills the information gaps and recommends practical solutions.

**Objective:**

The main aim of the study was to assess youth-friendly service quality and associated factors at public health facilities in Arba Minch town, Southern Ethiopia.

**Methods:**

Facility-based quantitative cross-sectional study supplemented with the qualitative design was conducted from September to December 2017 at two public health centers in Arba Minch town. Sample sizes of 403 young clients were included in the study using a systematic sampling technique. Data was collected by using an interview-administered questionnaire and observation checklist. Quantitative data analysis was made using SPSS version 20.0 to identify the association between the dependent and independent variables. Qualitative findings were coded and analyzed by using content analysis in Microsoft Excel. Finally, results are presented using narrations, tables, and figures.

**Results:**

A total of 403 youth-friendly service clients participated in the study. The overall score input, process, and youth clients' satisfaction was 54.41%, 42.0%, and 49.1%, respectively. Age (15-19) [AOR (95% CI) = 3.2 (1.4-7.8)], employment [AOR (95% CI) = 6.4 (2-17)], place of YFS [AOR (95% CI) = 0.35 (0.1-0.8)], frequency of visit [AOR (95% CI) = 0.03 (0.0-0.3)], waiting time [AOR (95% CI) = 0.02 (0.0-0.09)], and comfort with providers' sex [AOR (95% CI) = 0.07 (0.02-0.2)] were factors which are significantly associated with client satisfaction in this study.

**Conclusion and Recommendation:**

The study revealed that the overall quality of youth-friendly health service is below-set criteria (not good quality) in its all components, i.e., structural, process, and output. So, improvement of facility setup, client-provider interaction, and service sensitivity to all young groups and waiting time of services is essential.

## 1. Background

The world population is composed of 18% of adolescents (10–19 years) and 26% of young people (10–24 years) [1]. Most are healthy, but there is still substantial premature death, illness, and injury among adolescents. Illnesses can hinder their ability to grow and develop to their full potential. Alcohol or tobacco use, lack of physical activity, unprotected sex and/or exposure to violence can jeopardize not only their current health, but also their health as adults, and even the health of their future children [2].

Adolescents and young people ages 10 to 24 are the largest groups ever to be entering adulthood in Ethiopian history making up 30% of total population [3]. This group faces different health problems due to health services associated, social and cultural barriers [1].

WHO carries out a range of functions to improve the health of young people, including production of evidence-based guidelines to support health services and other sectors; making recommendations to governments on adolescent health and the provision of high-quality, age- appropriate health services for adolescents; documenting progress in adolescent health and development; and raising awareness of health issues for young people among the general public and other interested stakeholders [2]. Youth-Friendly Services (YFS) is one of WHO strategies for enhancing health services quality for adolescents which aimed at availing “Services that are accessible, acceptable, and appropriate for adolescents. They are in the right place, at the right price and delivered in the right style to be acceptable to young people [4].” Yet, evidence from both high- and low-income countries shows that services for adolescents are highly fragmented, poorly coordinated, and uneven in quality [5].

Limited evidence is available on the quality of youth-friendly service [6]. The existing literature focuses merely on assessing factors that affect YFS utilization and quality focusing on specific dimensions [1, 3]. Therefore, this study fills the gap of information on youth-friendly service quality level by assessing all dimensions and provides local evidence for context-specific decision making. In addition, it helps health care providers, policy makers, and different organizations to improve youth-friendly services program in public health facilities.

## 2. Methods and Materials

A facility-based quantitative cross-sectional study supplemented by qualitative design was conducted from September 01 to December 30, 2017, at Arba Minch town governmental health institutions. Arba Minch town is the capital of Gamo Gofa zone located at Southern Ethiopia.

### 2.1. Population

The source populations for the study were all young peoples between 10 to 24 years who visited the selected public health facilities in the town for youth-friendly health service and managers and service providers working at Arba Minch town public health centers. Study populations were young peoples between 10 and 24 years who visited selected public health facilities in the town for youth-friendly health service during the study period. In addition, health center managers and YFS providers were study participants for qualitative inquiries. Youth-friendly service clients with an emergency condition, critically ill, and adolescents who were under 15 years and came alone to the health facilities are excluded from the study due to the difficulty of getting information and consent from them.

### 2.2. Sample Size and Sampling Procedures

The sample size for the study was calculated using a single population proportion formula, considering the following assumptions. From the previous study, the overall youth client satisfaction to health service, 60.7% [7], 95% confidence level, 5% degree of precision, and 10% nonresponse rate. Finally, the calculated sample size was 403 youths. In addition, two health center managers for YFS assessment interview, two health centers for YFS facilities observation, and six client-provider interaction sessions (WHO recommends at least three observations per site) are used to collect qualitative data for this study.

The study was conducted at the two public health centers in the town, Arba Minch and Shecha Health centers, as they are the only facilities providing youth-friendly service in the town. Samples were allocated proportionally to two public health facilities based on the last three months, an average number of youth clients flow. Finally, systematic sampling was used to select individual clients.

### 2.3. Measurements

Adolescent- & Youth-Friendly Services are services that are accessible, acceptable, and appropriate for adolescents. They are in the right place, at the right price (free where necessary) and delivered in the right style to be acceptable to young people (the terms “adolescent-friendly health services” and “youth-friendly health services” are used interchangeably). Quality of care is a care which is effective, efficient, accessible, acceptable, equitable, and safe to service users (WHO). In this study, if the health center scores 75% and above of WHO Quality standard by combining the three quality assessment items for structure, process, and output/ satisfaction level, it was classified as “Good quality” or “Good standard of care” and if the the score is below 75%, it was classified as “Not good quality” or “Below the standard of care.” Structural quality is concerned with the availability of adequate service providers, facilities, information, essential drugs, equipment, and basic infrastructures. Process quality is related to client-provider interaction including privacy, good communication, education, and use of job aids, guidelines, and examination and treatment procedure according to the WHO standard. The output quality is concerned with youth clients' satisfaction level towards service provided at YFS centers.

Standard of care or services is care or services that are delivered in accordance with technical and practical guidelines or other evidence-based care protocols set by WHO and MOH. Satisfaction is the satisfaction of youth gained during service delivery. It is the young client view to care level gained that increases the likelihood of future youth-friendly health service. Level of satisfaction is a “proportions of clients” who were satisfied with the variables; representing by five-point Likert scale (1) very dissatisfied, (2) dissatisfied, (3) neutral, (4) satisfied, and (5) very satisfied was to be used. For the overall satisfaction level, those who were satisfied in greater or equal to factor mean score of the items (i.e., 3.24 as a cutting point in this study) were categorized under “satisfied” and those who were satisfied in less than factor mean score of the items were categorized as “dissatisfied.” The terms “dissatisfaction” and “unsatisfaction” are interchangeably used in this paper.

### 2.4. Data Collection Procedures

Both quantitative and qualitative data collection methods were employed to generate findings from service facility, service providers, and service users. The quantitative data was collected through structured client exit interview questionnaires with 13 satisfaction items.

Qualitative data was collected through an interview checklist, facility observation, and client- provider interaction score sheets where all of them have equal weights. Data collection tools were adapted from the WHO [8] and national guidelines. All interview and observation instruments were first designed in English and then translated to Amharic to ease its utilization. Items had good internal consistency and reliability coefficient of (alpha) 0.80, 0.83, and 0.81 for input, process, and output items, respectively.

The data from the youth client exit interview were collected by two nurses. Health centers manager interview, facility and client-provider interaction observations were conducted by one senior health officer. One health officer supervised all data collection process. All data collectors and supervisor were intentionally selected and used from other facilities/are not belonging to study health centers.

### 2.5. Data Quality Assurance

Data collectors were trained carefully on interview and observation procedure and question contents. The client interview questionnaire was pretested on 5% of the sample size in one of the health facility at Arba Minch Zuria district before use to check for consistency and errors. Close supervision was made on a daily basis to ensure completeness and consistency of each questionnaire and checklist. Data entry and cleaning was made carefully to avoid potential errors during analysis stages to assure data quality.

### 2.6. Data Processing and Analysis

Quantitative data coding, sorting, and entry were made using Epi info version 3.1.5 and exported to SPSS version 20.0 statistical software for analysis. The analysis includes descriptive statistics which was computed for the study sociodemographic and other explanatory variables. Quantitative data analysis was made using SPSS version 20.0 to identify the association between the dependent and independent variable. Finally, the results of quantitative data were presented using text, tables, and charts. Qualitative data were sorted, coded, and analyzed by using content analysis method in Microsoft Excel, 2010 and the results are presented in the form of narratives in three main parts based on Donabedian quality of care model, the structure-process-output.

### 2.7. Ethical Considerations

Ethical clearance was obtained from Arba Minch University Institutional board review committee (IBRC). And then, official permission to cascade data collection was handed over from respective local authorities including Arba Minch town health offices and Arba Minch and Shecha health centers. During data collection time, informed oral consent was taken from each study participant. Moreover, confidentiality was maintained through anonymity and privacy measures to protect respondent's right through the research process.

## 3. Result

### 3.1. Sociodemographic Characteristics of Study Participants

A total of 403 youth-friendly service clients participated in the study, with response rate of 100%. Among them, 52.6% were males, while 47.4% are females. The age of the respondents ranges from 10 to 24 with mean and standard deviation of 19 and 3, respectively. Most of the YFS clients (79.2%) are single, 52% of them are from secondary & preparatory school levels and 47.9% are Gamo in their ethnicity. Moreover, most of youth segment that visited YFS centers (72.2%) were found to be unemployed (Table 1).

### 3.2. The Structural Quality of Youth-Friendly Health Service

The health centers have a total of 80 health workers. Youth-friendly services are provided by trained (YFS) health workers. But there is inadequate health workers training (8.7%) and assignment of trained health workers (only two per site). All service providers are found to be females with the age ranging from 25 to 40 years (Table 2).

All facilities have separate YFS rooms and adequate medical instruments which enable them to provide minimum packages. Shecha health center has no waiting area. But two of HCs faced an interruption of essential supplies like HIV kit and condoms in the past 12 months. Information, education, and communication (IEC) materials to educate youth clients are not available. Two of the health centers have transportation (ambulance) and communication (telephone), electricity, running water, functional toilet, and waste disposal facilities.

Service information delivering materials like sign post is erected at both YFS sites, but list of service hours and service provided to young clients is not posted at Shecha health center. Service opening hour for two of YFS facilities is from Monday to Friday (from 2:30 to 11:30 local time) and YFS corners are closed at night time and weekends. Arba Minch health center provides all of minimum packages of YFS recommended by World Health Organization. But Shecha health center does not provide postabortion care, due to lack of trained manpower. Facilities have no system involving youth in YFS program. They were not engaged in planning, implementing, and evaluating services. None of health centers included youth in their governance structure, i.e., board of health centers.

**Figure 1:**
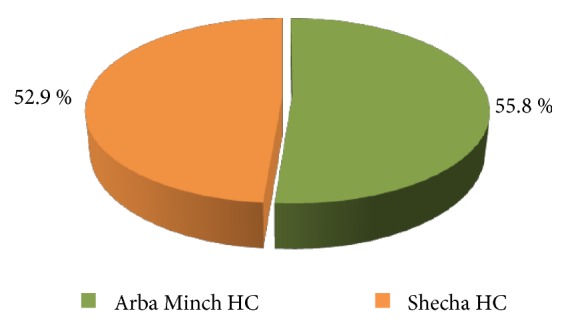
Percentage of good score of structural quality of youth-friendly service at public health facilities, Arba Minch, Southern Ethiopia, December 2017.

Overall, the percentage of good score of YFS structural quality is 54.41%, and the two health facilities rating point shows slight variation, where Arba Minch health center (55.8%) has relatively better score than Shecha health center (52.9%) (Figure 1).

*Note*. Percentage is computed from total number of good score (19 and 18 out of 34 for Arba Minch and Shecha health center, respectively)*∗*100 divided by total number of items for both facilities.

### 3.3. The Process Quality of Youth-Friendly Health Service

Findings of client-provider interaction show that none of providers introduced themselves to clients to build good rapport. Only in 16.6% cases providers asked about their psychosocial history, and in cases (66.6%) provider listened to clients with attention.

Regarding privacy, in some cases (33.3%), providers assured clients about confidentiality issues. In most cases (66.6%), auditory and visual privacy is secured. In line with this, only 33.3 % of cases are asked permission before physical examination. Interruptions were common at Arba Minch health center.

During treatment provision, half (50%) of consultation providers had sufficient time to deal with clients, and accurate information on medical condition, risk reduction and prevention methods, and treatment options is provided in 66.6%, 66.6%, and 83.3%, cases, respectively. Observations also identified that providers give less focus, time, and information to clients who receive illness related (non-RH) services. Audio-visual material to educate clients is not used at all cases. All of (100%) clients are provided with the requested service without any denial and 50% of cases were informed about the services available to youth clients. The process quality good score is higher for Shecha health center (53.97%) than that of Arba Minch Health center (30.16%), and overall percentage of good score for two facilities is 42 % (Table 3).

### 3.4. The Output Quality of Youth-Friendly Health Service (Youth Client Satisfaction)

#### 3.4.1. Service Use and Experiences

**Figure 2:**
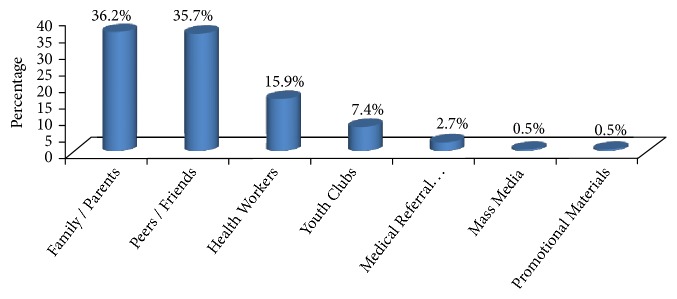
Source of information for visiting health facilities, Arba Minch, Southern Ethiopia, December 2017.

Above half of clients, 254 (63 %) have previous experience of visiting health centers. Among those who visited health facilities before, most (246, 96.5%) have served in different frequencies in the past 12 months. Of them, 48.8%, 44.7%, and 6.5% visited one time, two to four times, and five and more times, respectively. Regarding information about youth-friendly service, family members (36.2%) and peers/friends (35.7%) become the major sources. Others like health workers and youth clubs account 29% of information sources (Figure 2).

**Figure 3:**
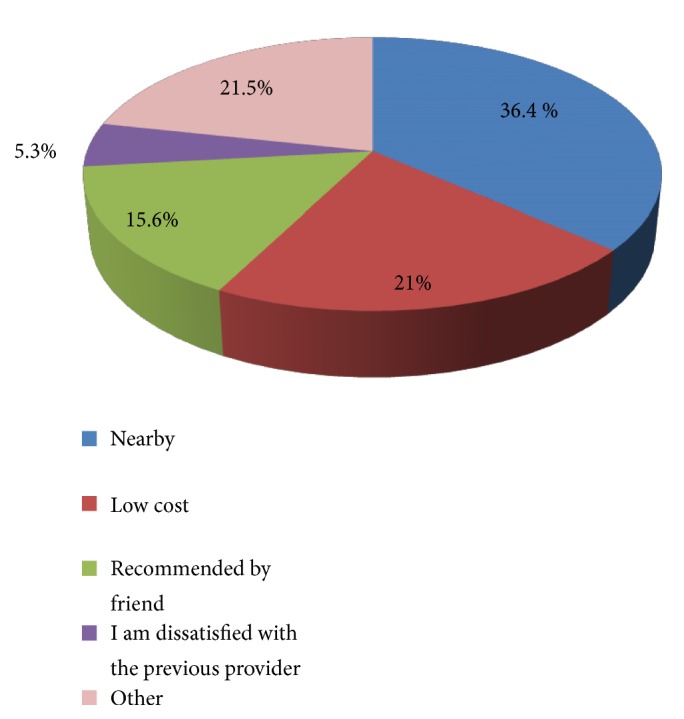
Reasons of clients to choose youth-friendly health facilities, Arba Minch, Southern Ethiopia, December 2017.

Regarding reasons of seeking services in the YFS facilities, most respondents replied as because it is nearby facility (36.4 %) and low cost of services (21%) (Figure 3). As this is more evidenced in terms of distance/time taken to reach the facilities; most (70.2%) travelled for less than 30 minutes; others (27.3%) travelled from 30-minute to 1-hour period and 2.5% travelled more than one hour to reach the center. Regarding length of waiting time, around 18.5% of clients waited for more than one hour and (49%) waited for 30 minutes to 1 hour and 32.5% waited for less than 30 minutes to get service after reaching health facilities.

Regarding services they received from youth-friendly health corners, most of the youth had used services related to treatment of other medical conditions (44.6%), HIV testing and counseling (33.3%), and other like clinical and nonclinical services accounting for 20% (Table 4).

Most clients (95%) received all services they wanted on the day of their visit. However, 5% of them did not get all services they demanded. The reasons for missing services were feeling discomfort to request services (30%), service unavailable on the day of visit (20%), the provider did not have time (20%), and client-related inconveniences: urgency of clients (10%) and lack of knowledge about the service availability (5%) and others accounting for 15%. In line with this, all clients perceive that YFS center is open to all youth groups, but 63.2% respondents are not comfortable with sex of service providers.

Majority (91%) of respondents said that they will recommend the services to their friends and 93.5% responded that they will revisit to service delivery point in the future.

#### 3.4.2. Youth Client Satisfaction

About 198 (49.1%) of clients were satisfied with the care given in youth-friendly health service corner with mean of 3.24 as cutting point. Level of satisfaction for clients who visited Shecha health center is 60.7% and that of Arba Minch health center is 43.3%.

Around 43.9% males and majority (55%) of females are satisfied. Satisfaction for different age is 56.2 %, 54.7 %, and 41.9 % for youth clients of 10-14, 15-19, and 20-24 age groups, respectively. Sixty-six percent of employed clients and 42.6 % of unemployed ones are satisfied and the rest are dissatisfied with services.

**Figure 4:**
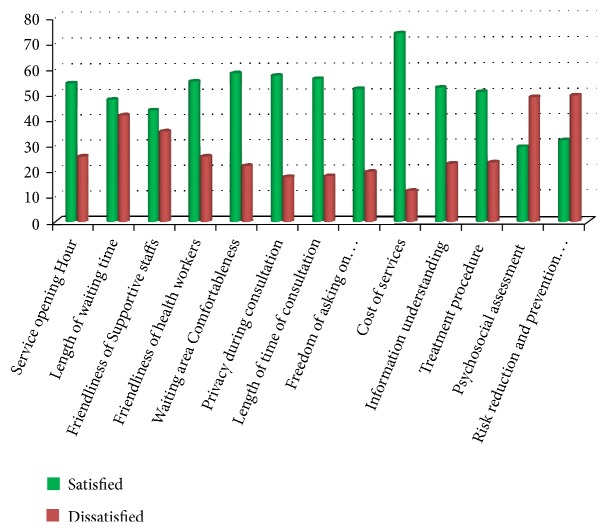
Percentage of youth client satisfaction towards various YFS dimensions at public health facilities, Arba Minch, Southern Ethiopia, December 2017.

Regarding service characteristics, most of respondents are satisfied with cost of service (73.9%), and satisfaction to service opening hour, friendliness of staffs, privacy is within range of 50% to 60%. Low satisfaction is responded to questions related to level of satisfaction on adequacy of psychosocial assessment (29.5%) and information given on risk reduction and prevention (32%) (Figure 4).

*(1) Factors Associated with Youth Client Satisfaction. . *After being adjusted for important covariates in a multivariable model, the variables that independently predict youth client satisfaction were age (15-19) [AOR (95% CI) = 3.2 (1.4-7.8)], employment [AOR (95% CI) = 6.4 (2-17)], place of YFS [AOR (95% CI) = 0.35 (0.1-0.8)], frequency of visit [AOR (95% CI) = 0.03 (0.0-0.3)], waiting time [AOR (95% CI) = 0.02 (0.0-0.09)], and comfort with providers' sex [AOR (95% CI) = 0.07 (0.02-0.2)] (Table 5).

Age is significantly associated with YFS satisfaction, in that respondents within the age group of 15-19 were 3.2 times more likely to be satisfied with YFS compared to those within the age group of 20-24 years [AOR (95% CI) = 3.2 (1.4-7.8)]. Employment is also associated with YFS satisfaction; employed clients were 6.4 times more likely to be satisfied with YFS compared to employed clients [AOR (95% CI) = 6.4 (2-17)]. Place of YFS is one of the predictors. Clients who visited Arba Minch HC YFS are 65% less likely to be satisfied than those who visited Shecha HC [AOR (95% CI) = 0.35 (0.1-0.8)]. Waiting time is significantly associated with youth satisfaction. Clients who waited for services for more than one hour are 98% less likely to be satisfied with YFS compared to those who waited for less than thirty minutes [AOR (95% CI) = 0.02 (0.0-0.09)]. Comfort with providers' sex is the other associated factor. Clients who are not comfortable with providers' sex are 93% less likely to be satisfied with YFS compared to those who are comfortable with providers' sex [AOR (95% CI) = 0.07 (0.02-0.2)].

### 3.5. Overall Quality of Youth-Friendly Health Services

The overall score and level of YFS for input, process, and output quality dimension is 54.41%, 42.0%, and 49.1%, respectively. The process quality is the most compromised dimensions among them. As observed in the table below, all of YFS sites scored lower than the set cutoff point (75%) in three of quality dimensions. Hence, the overall quality of YFS is categorized as “not good quality” or “below standard” (Table 6).

## 4. Discussion

The overall level of quality for youth-friendly service for structural, process, and output dimension is 54.41%, 42.0 %, and 49.1%, respectively. All of YFS sites scored lower than the set cutoff point (75%) in three of quality dimensions and the overall quality of YFS is categorized as “not good quality” or “below standard.” It is consistent with quality assessment report from Tanzania [9] and lower than the study conducted in China [10].

The level of structural quality is poor and it is comparable with YFS assessments made at Uganda [11]. On the other hand, it is lower or poor quality when compared to findings of South Africa and China [10, 12]. It is not in line with WHO YFS standard set for service accessibility and acceptability criteria [13]. This could be due to lack of resources and competing health priorities in the study area.

Similarly, process indicators are not in line with WHO YFS standard set for service effectiveness criteria [13] and are consistent with findings at Uganda and Mongolia [11, 14]. It is very poor in quality when compared to the studies conducted in Egypt [15]. This is may be the result of difference in health care system related to providers training, competency, and specialization. However, both structural and process qualities in Arba Minch town is found to be better than that of findings in Ghana and Botswana, where poor clients-provider interaction and competencies of HW (untrained YFS providers) were observed in later case [16]. This could be due to the existence of nongovernmental organizations support in the study area.

In this study, age is significantly associated with YFS satisfaction, in that respondents within the age group of 15-19 were 3.2 times more likely to be satisfied with YFS compared to those within the age group of 20-24 years. This is in line with the study conducted in Germany, where younger adolescents showed higher satisfaction than older [17]. Decrease in satisfaction with age may in part be due to the fact that older adolescents have increased health concern, achieve growing understanding and cognitive skill, begin to frame their independent views about their social environment, and are encouraged to develop their own points of views. In contrary, a study conducted in Mongolia shows lesser satisfaction among younger adolescents. This may be due to variations in service expectation, type of service demanded and provided [14].

Employment is also associated with YFS satisfaction; employed clients were 6.4 times more likely to be satisfied with YFS compared to employed clients. As stated in Serbia findings, unemployed patients tend to estimate their health condition as worse and are preoccupied with perception that service quality provided to them will be poor, which will create communication barrier with health workers [18]. However, study conducted in America revealed that employed clients tend to have lower satisfaction than their counterparts. This difference could be due to difference in client attitude, provider practice, and expectation of service quality [19].

Waiting time is significantly associated with youth satisfaction. Clients who waited services for more than one hour are 98% less likely to be satisfied with YFS compared to those who waited for less than thirty minutes. This finding is supported by study conducted in Ethiopia, Amhara region, which indicates that clients who waited longer time are more dissatisfied as compared to their counterparts [20]. However, the finding is not consistent with the study conducted in China [10]. This may be attributed to the low service utilization, proportional number of health care providers with clients, and awareness of study participants to understand that some health care service requires time to provide quality of care.

Comfort with providers' sex is the other associated factor. Clients who are not comfortable with providers' sex are 93% less likely to be satisfied with YFS compared to those who are comfortable with providers' sex. Different reports entail about the relation of sex of service providers and that of clients and its association with satisfaction. In one study conducted in Nepal, most of patients reported that they are not happy with sex of health workers and are not satisfied with services provided to them [21]. This is because of the fact that those clients who do not meet their sex preferences are often in difficulty telling the story, take time during consultation, and maintain informational partnership and trust.

The overall youth client satisfaction, output quality, is 49.1%. This is comparable with findings of the study conducted at Iran Kerman province (49.6%) [22] and Ethiopia at Dessie town (58.9%) [23]. However, the level of satisfaction is higher than that of Serbia (42.8%) [18] and Ethiopia West Amhara region (39.3%) [20]. This may be due to the difference in study population (hospitalized patients included at study made in Serbia) and commitment of health care managers and service providers as explained in those studies.

## 5. Limitation of the Study

- The study is geographically limited to Arba Minch town and it focused only on clients who visited public health facilities; it cannot be generalized to clients who might not come to health institutions and clients who use private settings.
- In measuring each of the quality components, overestimations of findings may happen due to Hawthorn effect, interobserver bias, and courtesy or social desirability biases specially in measuring process quality.
- The study also shares the limitation of cross-sectional design as in this case it is difficult to establish the temporal sequence between independent and outcome variable.

## 6. Conclusions

The study revealed that the overall quality of youth-friendly health service is below WHO standard in terms of its three components, i.e., structural, process, and output qualities. The structural quality is affected by lack of waiting areas, insecure supplies, and absence of IEC-BCC materials and poor system of planning and monitoring YFS services. Inadequate privacy and poor communication and nonuse of job aids hindered the process quality.

## 7. Recommendation

Based on the study findings, the following recommendations are made.

*To Health Workers Who Provide YFS*

- Privacy and confidentiality should be well secured by changing the practice of providers (e.g., minimizing interruptions during client visits).
- The health workers who provide YFS shall give attention to time and content (psychosocial history and information about the medical condition) of consultation during client-provider interaction.

*To Health Care Managers at All Levels*

- They should make service delivery points friendlier by dealing with providers and improving opening hours.
- Longer waiting time to get service shall be reduced by assigning adequate number of providers and increasing service delivery points to make service more accessible. Moreover, awareness should be given to clients regarding nature (time) of services requested.
- Structural inputs like building waiting area (Shecha HC), minor renovations of consultation rooms, and availing IEC materials should be done at YFS centers.
- The logistics of commodity and drug supplies should be strengthened to ensure that condoms and STI drugs are available to young people when they need them.
- Health centers shall involve youth in planning, implementation, and evaluation phase in youth-friendly service.

(vi) In addition, further studies focusing on factors that affect youth service utilization in Arba Minch town are needed.

## Figures and Tables

**Table 1:** Sociodemographic characteristics of study participants in Arba Minch youth-friendly service, Sothern Ethiopia, December 2017 (N=403).

Variables	Category	Frequency	Percentage
Age	10-14 years	32	7.9
	15-19 years	192	47.6
	20-24 years	179	44.4
Education status	Illiterate	30	7.4
	Primary school	80	19.9
	Secondary & preparatory school	210	52.1
	College and above	83	20.6
Marital status	Single	319	79.2
	Married	59	14.6
	Others^*∗*^	25	6.2
Religion	Orthodox	151	37.5
	Muslim	60	14.9
	Protestant	162	40.2
	Other^*∗∗*^	30	7.4
Current	Daily laborer	49	12.2
occupation	Private business /trader	34	8.4
	Commercial sex worker	14	3.5
	Others	15	3.7
	Unemployed	291	72.2
Ethnicity	Gamo	193	47.9
	Gofa	87	21.6
	Amhara	94	23.3
	Other*∗∗∗*	29	7.2

*∗* Widowed and separated *∗∗*Catholic *∗∗∗* Wolayita, Zayse, Gidicho, and Oromo

**Table 2:** Percentage of health professionals who received YFS packages related trainings, Arba Minch, Southern Ethiopia, December 2017.

Name of health center	Total number of health workers	Number of health workers trained by type of services									
		YFS*∗*		PAC*∗*		LAFP*∗*		VCT/PITC/HCT*∗*		STIs*∗*	

	Number	No.	%	No.	%	No.	%	No.	%	No.	%

Arba Minch	46	5	10.8	2	4.3	5	10.8	13	28.2	13	28.2
Shecha	34	2	5.8	0	0	8	23.5	10	29.4	2	5.8
Total	80	7	8.7	2	2.5	13	16	23	28.7	15	18.7

*∗*YFS: youth-friendly service, PAC: postabortion care, LAFP: long-acting family planning, VCT: voluntary, counseling, and testing, PICT: provider initiated counseling and testing, HCT: HIV counseling and testing, STI: sexually transmitted infection.

**Table 3:** Process quality indicators of youth-friendly service, Arba Minch, Southern Ethiopia, December 2017.

Process quality indicators	Percentages (%) of good scores	
	Arba Minch	Shecha
	Health Center	Health Center
Visual privacy	0.0	66.6
Auditory privacy	33.3	100
Provider introduced him/herself	0.0	0.0
Assured confidentiality	66.6	0.0
Provider listened to client with attention	66.6	66.6
Measured vital signs	66.6	66.6
Asked about psychosocial history	0.0	33.3
Used job aids and case management guides	0.0	33.3
Provide sufficient time for consultation	0.0	100
Permission before physical examination	33.3	33.3
Provide information on medical condition	66.6	66.6
Provide information on treatment options	66.6	100
Ask client preference for treatment options	33.3	66.6
Provide information on risk reduction and prevention Methods	33.3	100
Use audio-visual materials	0.0	0.0
Provide information on follow-up actions	33.3	66.6
Informs the service available to clients	33.3	66.6

**Table 4:** Services utilized by youth-friendly service clients, Arba Minch, Southern Ethiopia, December 2017.

Type of services	Clients utilized services (n=403)	
	Yes	No
Illness-related condition^*∗*^	273	130
HIV counseling & testing	204	199
Entertainment services	67	336
FP services	19	384
IEC-BCC services	16	387
STI service	10	393
Violence-related service	6	397
Safe abortion service	3	400
Maternal health services	2	401
Other services	11	392

*∗*Illness-related conditions are medical conditions other than reproductive health problems.

**Table 5:** Factors associated with youth client satisfaction towards YFS at public health facilities, Arba Minch, Southern Ethiopia, December 2017.

Variables	Satisfaction level		COR (95% CI)	AOR (95% CI)
	Satisfied	Unsatisfied		
*Sex *				
Male	93	119	0.6 (0.4-0.9)	
Female	105	86	1	

*Age*				
10-14	18	14	1.8 (0.8-3.8)	2.5 (0.6-11)
15-19	105	87	1.7 (1.1-2.5)	3.2 (1.4-7.8)^*∗*^
20-24	75	104	1	1

*Employment*				
Employed	74	38	2.6 (1.6-4)	6.4 (2-17)^*∗*^
Unemployed	124	167	1	1

*Length of waiting time*				
More than one hour	14	61	0.2 (0.09-0.3)	0.02 (0.0-0.09)^*∗*^
30 minutes to 1 hour	108	90	0.8 (0.5-1.3)	1 (0.3-2.6)
Less than 30 minutes	76	54	1	

*Get service for illness-related condition*				
Yes	127	146	0.7 (0.5-1.0)	0.4 (0.2-1.0)
No	71	59	1	1

*Sex of service providers*				
Comfortable	104	44	1	1
Not comfortable	94	161	0.2 (0.2-0.9)	0.07 (0.02-0.2)^*∗*^

*∗*Significant at p value <0.05

**Table 6:** Quality of youth-friendly service at public health facilities, Arba Minch, Southern Ethiopia, December 2017.

Quality components	Percentage scored by youth-friendly service facilities	
	Arba Minch Health Center	Shecha Health Center
Structural quality	55.88%	52.94%
Process quality	30.16%	53.97%
Output quality (satisfaction)	43.3%	60.7%

## Data Availability

The data used to support the findings of this study are available from the corresponding author upon request.

## Abbreviations

AIDS: Acquired immunodeficiency syndrome
HC: Health center
HIV: Human immunodeficiency virus
HW: Health worker
ICT: Information and communications technology
ICPD: International Conference on Population and Development
IEC: Information, education, and communication
LAFP: Long-acting family planning
LMICs: Low- and middle-income countries
MOH: Ministry of Health
OPD: Outpatient department
PAC: Postabortion care
RH: Reproductive health
SPSS: Statistical Package for Social Sciences
STI: Sexually transmitted infections
SRH: Sexual and reproductive health
VCT: Voluntary, counseling, and testing
WHO: World Health Organization
YFS: Youth-friendly service.
